# Autologous Posterior Rectus Sheath as a Vascularized Onlay Flap: a Novel Approach to Hiatal Hernia Repair

**DOI:** 10.1007/s11605-021-05134-7

**Published:** 2021-09-10

**Authors:** Yalini Vigneswaran, Ava F. Bryan, Brian Ruhle, Lawrence J. Gottlieb, John Alverdy

**Affiliations:** Sections of Minimally Invasive Surgery and Plastic Reconstructive Surgery, Department of Surgery, University of Chicago Medical Center, 5841 S Maryland Ave, MC5095, Chicago, IL 60637 USA

**Keywords:** Paraesophageal hernia, Rectus flap, Hiatal repair

## Abstract

**Introduction:**

Complex and recurrent paraesophageal hernia repairs are a challenge for surgeons due to their high recurrence rates despite the use of various prosthetic and suturing techniques.

**Methods:**

Here we describe the use of vascularized fascia harvested from the posterior rectus sheath with peritoneum during robotic hiatal hernia repair in two patients with large complex diaphragmatic defects.

**Results:**

Successful harvesting and onlay of the right posterior rectus sheath based on a falciform vascular pedicle was achieved robotically by rotating and securing the flap to the diaphragmatic hiatus as an onlay flap following cruroplasty of the hiatal defect.

**Conclusions:**

In patients with difficult to repair large paraesophageal hernias, we demonstrate a promising new technique to restore the dynamic hiatal complex with the tensile strength of autologous vascularized fascia and peritoneum.

**Supplementary Information:**

The online version contains supplementary material available at 10.1007/s11605-021-05134-7.

## Introduction

Despite the use of various prosthetic materials and advances in suturing techniques by high volume esophageal surgeons in specialized centers, the long-term success of hiatal hernia repair in older patients with large hiatal defects remains a challenge. Hiatal hernia surgery violates one of the major tenants of hernia surgery given that it involves approximating muscle, not fascia, and often does not result in a tension free repair. It may be for this reason that primary laparoscopic repair continues to carry unacceptable long-term recurrence rates of up to 50%.^1,2^ The need to reinforce the hiatus with durable material to counteract any resultant tension has taken two primary forms: reinforcement with permanent mesh or reinforcement with biologic mesh. Both of these approaches have resulted in suboptimal long-term results compared to simple primary suture repair.^3,4^ Furthermore, permanent mesh carries an unacceptable risk profile of shrinkage with erosion into the esophageal lumen, dense fibrosis, and esophageal stenosis which can lead to disabling dysphagia.^5–7^ Biologic mesh avoids many of these complications but does not appear to serve any benefit for long-term recurrence rates and is clinically equivalent to primary repair without mesh with added costs.^8–10^ Finally, reoperative surgery in patients in whom prosthetic material has been placed or in whom prosthetic material still remains fraught with major complications and is often avoided despite patients suffering large recurrences associated with disabling symptoms.

**Fig. 1:**
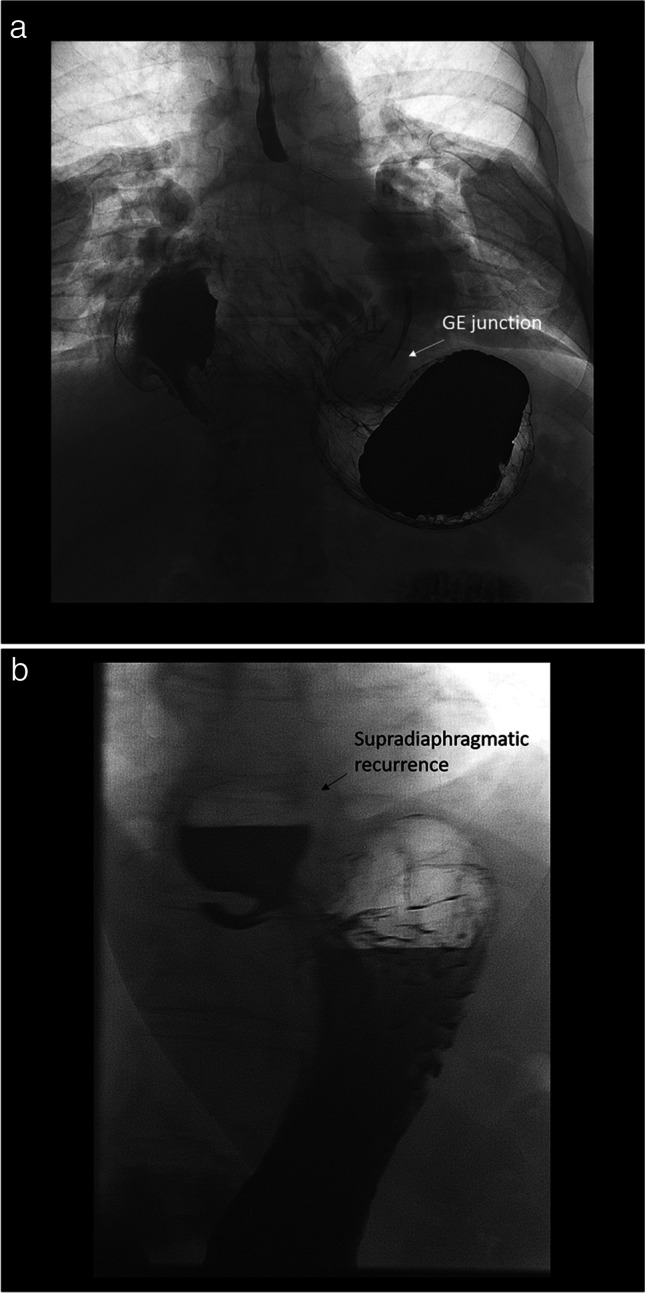
**a** Patient 1 with large paraesophageal hernia and gastric volvulus. **b** Patient 2 with recurrent paraesophageal hernia

Here we describe a novel use of an autologous vascularized posterior rectus sheath fascia onlay to reinforce hiatal hernia repair via the robotic approach. As recently described for complex abdominal closure following liver transplant, we employed similar principles of harvesting a posterior rectus sheath flap^11^ that remained vascularized via the falciform ligament of the liver that we verified by indocyanine green (ICG) injection and imaging. The vascularized segment of the right posterior rectus sheath is rotated to the diaphragmatic hiatus after suture approximation of the hernia defect, circumferentially fashioned around the esophagus and sutured to the diaphragm. A major advantage of this approach is the use of an autologous high integrity and thick tissue including the peritoneum that remains vascularized and properly orientated such that the peritoneum is facing the stomach. The herein described approach during robotic surgery is straightforward and simple and offers the advantage that the vascularity of the transposed flap can be confirmed with ICG, the peritoneum is facing the stomach possible lessening the effect of scarring and adhesions, and the muscle side of the graft faces the diaphragm. The patients’ characteristics are described here:

Patient 1 is a 69-year-old man with chronic large paraesophageal hernia with mixed type gastric volvulus (Fig. 1a) which was increasingly symptomatic. He had no previous surgery and was otherwise in good health with no significant co-morbidities. His hiatal hernia had been known to be present for over 10 years during which he contemplated surgery based on intermittent episodes of partial obstruction. Patient is now 5 months postoperatively from the described technique and asymptomatic with no further episodes of pain, vomiting, obstruction, or dysphagia.

Patient 2 is a 72-year-old woman with a recurrent paraesophageal hernia. She had her initial laparoscopic repair with biologic mesh and a partial (270°) posterior fundoplication approximately 18 months prior which failed 3 months after surgery based on recurrent symptoms of abdominal pain, GERD symptoms, and imaging showing a recurrent hernia (Fig. 1b). We hypothesize this recurrence was multifactorial but likely due to patient’s age, size of the original defect, attenuated tissues, and tension. The patient is now 5 months postoperatively from the described technique and asymptomatic with no further episodes of pain or GERD.

## Operative Technique

**Fig. 2:**
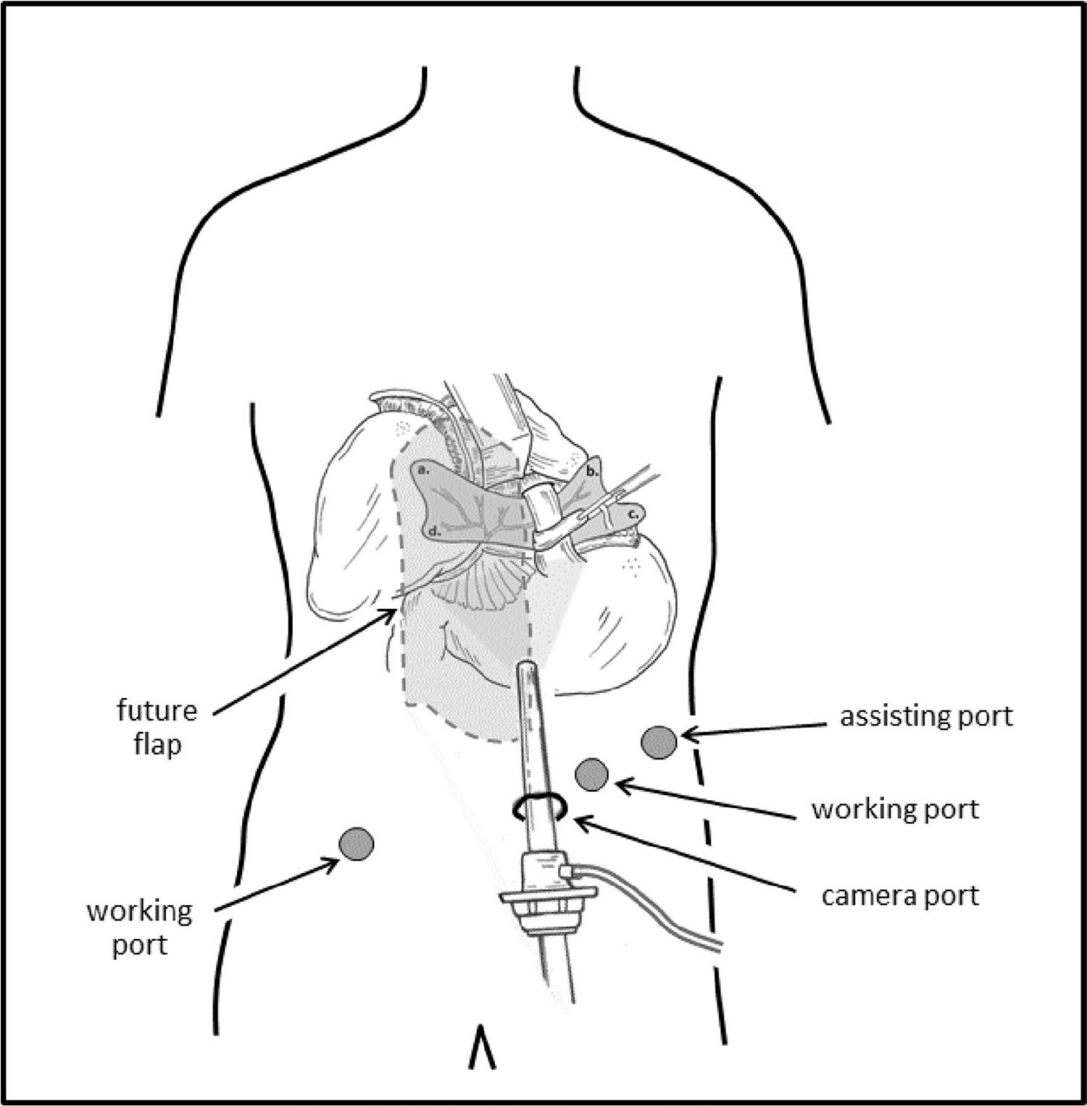
Adjusted port placement to allow for easy robotic assisted harvest of the right posterior rectus sheath but still allowing for hiatal hernia reduction and repair

Robotic hiatal hernia repair has been described by others^12–14^ and is carried out in standard fashion with total sac excision, intra-abdominal mobilization of the distal esophagus, suture repair of the hiatal defect by approximating the crura, and performance of an anti-reflux fundoplication. Once this is carried out and completed, harvest of the right posterior rectus sheath with its blood supply is achieved via preservation of the falciform ligament of the liver. First, the laparoscope angle is changed to 30° up. The only deviation from the original reports of robotic hiatal hernia repair is that ports are placed well below the umbilicus to allow for access to the intended right posterior rectus sheath flap (Fig. 2). Additionally the Nathanson liver retractor is placed far left of midline to avoid disruption of the falciform ligament and the vascular pedicle.

**Fig. 3:**
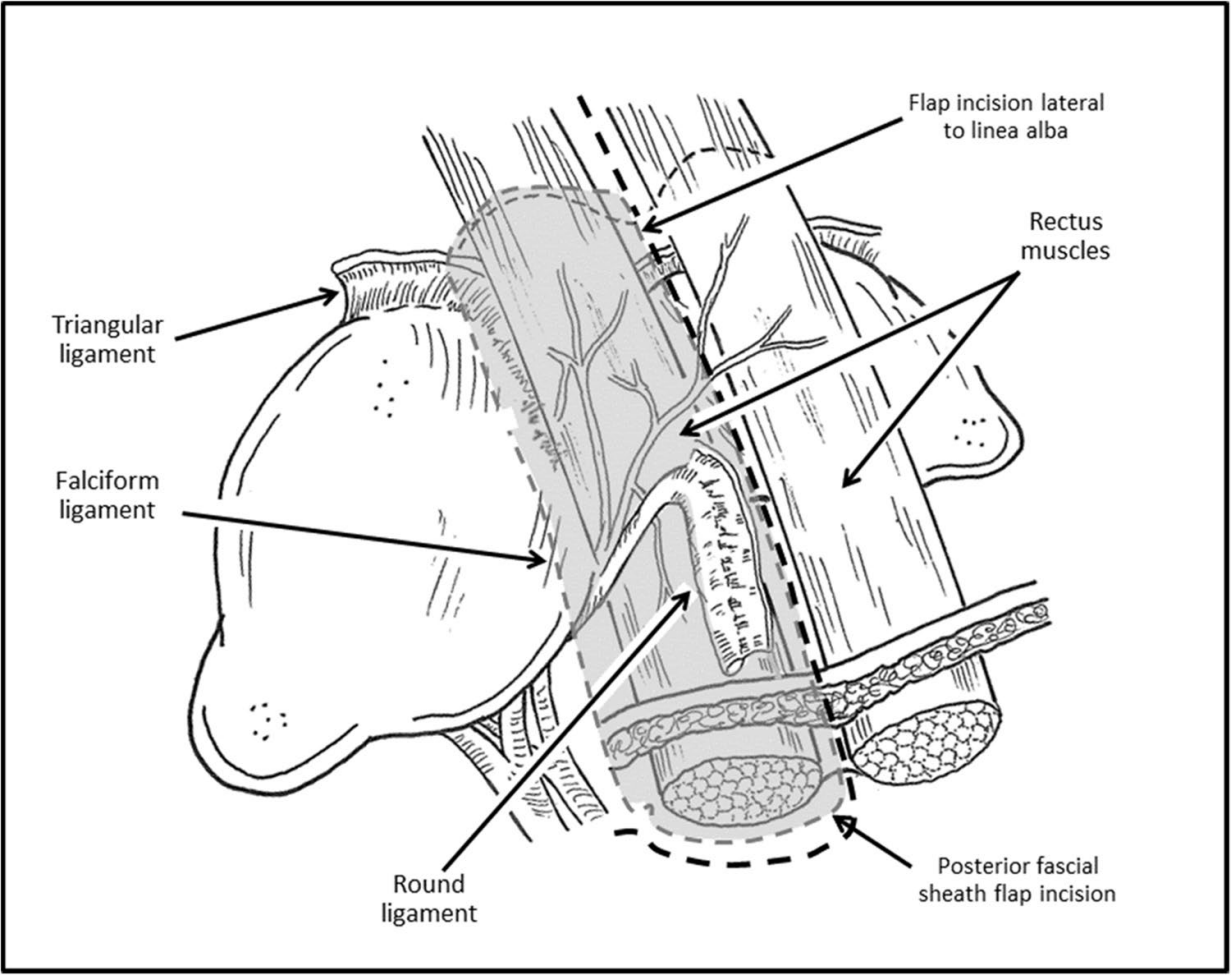
Harvesting the right posterior fascia flap with preservation of draining vessels within the falciform and round ligaments

The dissection proceeds using the robotic scissors with cautery function and begins laterally at the site at which the lateral line of the rectus fascia/muscle is visualized. A posterior rectus sheath segment of approximately 12 cm in the longitudinal axis and 8 cm in the horizontal axis is envisioned to be harvested while remaining attached to the falciform and round ligaments. Cautery marks can be used to map out the projected tissue harvest. Beginning laterally (longitudinally) and caudally (horizontally), a plane is developed until which time the rectus muscle is visualized indicating that the proper plane has been entered. This incision is extended laterally to the lateral edge of the rectus muscle and medially just lateral to the linea alba (Fig. 3).

**Fig. 4:**
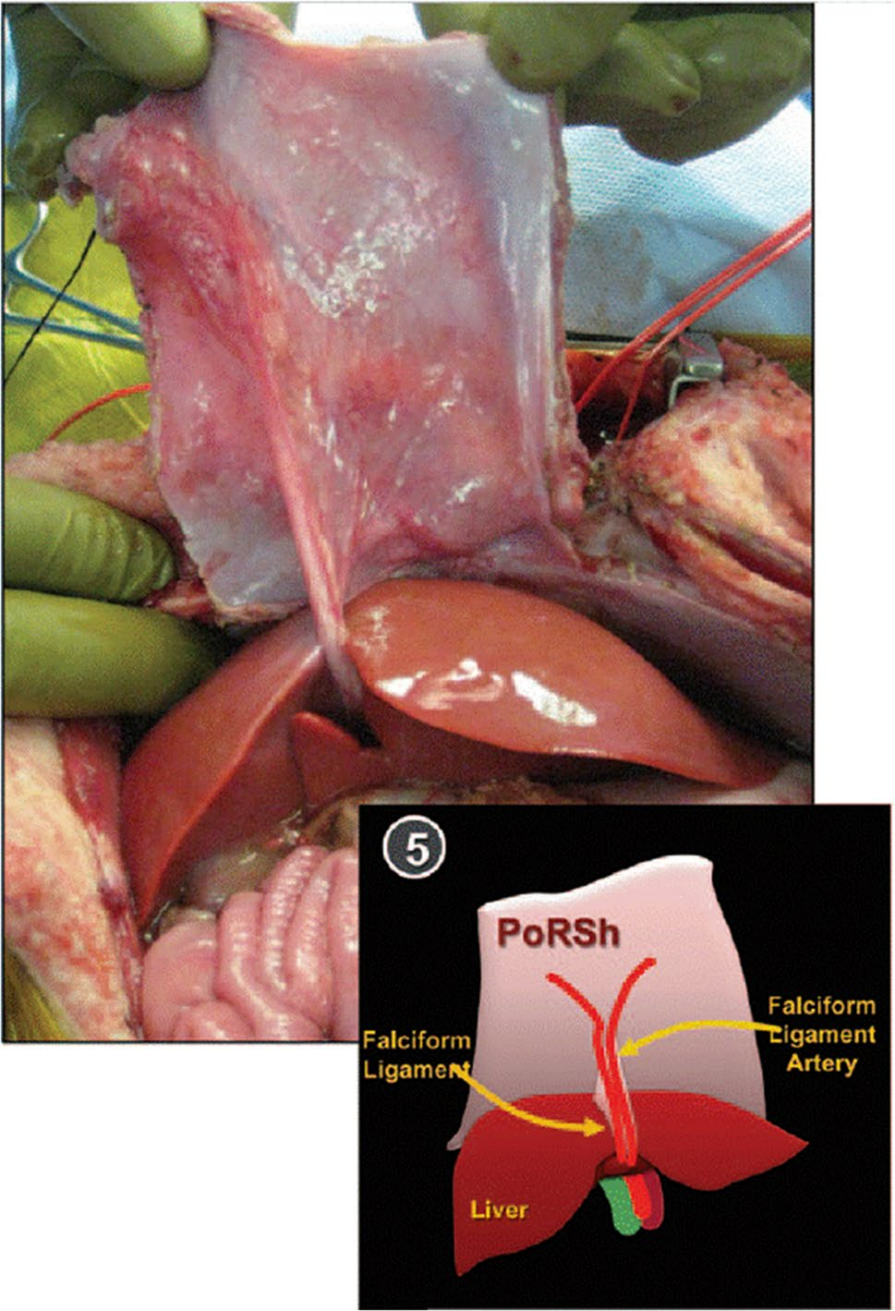
Vascularized flap based on the falciform ligament as first described by Gottlieb et al.^15^ Note the thickness of the harvested posterior rectus sheath (PoRSh) flap and its attachment to the round ligament of the liver. The blood supply of the flap is provided by the hepatic falciform artery, and its integrity can be verified via ICG injection

During this dissection, it is important to preserve the integrity of the linea alba but with careful preservation of the round ligament and its associated vessels which maintain the vascularity of the flap. This dissection of the posterior sheath is then carried cephalad toward the falciform ligament. Upon approaching the falciform ligament, the cephalad edge/border of the flap is designated by opening the avascular portion of the falciform ligament, while preserving the draining vessels through the falciform. The dissection of posterior sheath is then completed, with the sheath free from the muscle and solely attached by the falciform ligament. Once the graft/flap is fully mobilized and attached only by the falciform ligament (Fig. 4), the indocyanine green (ICG) 5 mg dose is injected, and the vascularity examined (see technique video).

**Fig. 5:**
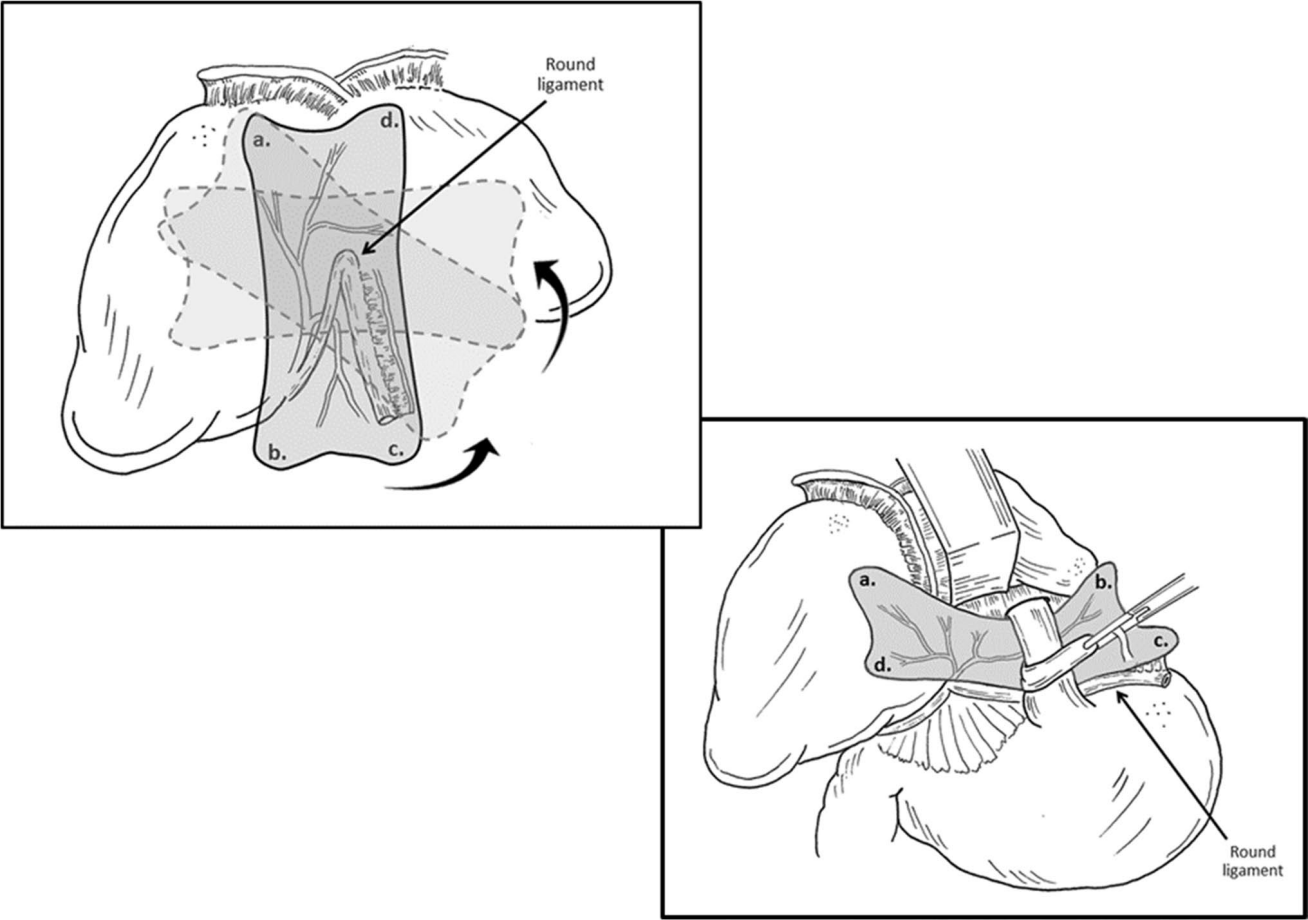
**a** Native flap location with the future rotation from the anterior abdominal wall, counterclockwise 90°. **b** Flap placement up to the hiatus with the antiperitoneal side placed against the diaphragm. Corners of flap labeled to orient the rotation of the flap towards the hiatus

The flap is then rotated under the liver allowing the falciform ligament to fall within the sagittal fissure. The flap is brought under the left lobe of the liver without twisting of the pedicle towards the hiatus (Fig. 5a) making sure that the peritoneum is facing the stomach. At this point, a keyhole-like incision can be made at the surgeon’s discretion to allow the graft to encircle the esophagus. NOTE: the posterior sheath flap, in contrast to prosthetic material, can be placed snug against the esophagus given that it is autologous vascularized tissue with a low potential to fibrous and constrict the esophagus. Keeping the peritonealized side of the flap intra-abdominal, the sheath is attached to the diaphragmatic hiatus with as many sutures as needed to affix it against the defect and diaphragm such that it appears flat and partially stretched along the diaphragmatic muscle while avoiding key vascular structures on both the right (vena cava) and left (right ventricle) sides (Fig. 5b).

*Technique video link:* https://www.dropbox.com/s/v5jjobub76662av/Posterior%20rectus%20sheath%20PEH%20repair%20Vigneswaran.mov?dl=0

## Discussion

### Falciform Ligament

Due to the location, length, and mobility of this vascularized tissue, the falciform ligament can be mobilized and has been used for various purposes. The falciform attaches to the anterior abdominal wall in continuity with the parietal peritoneum of the diaphragm and posterior rectus sheath. This pedicle is supplied primarily from the liver and either the left or middle hepatic artery forming a network of vessels which runs through the broad falciform ligament perfusing the posterior rectus and overlying peritoneum; this is also referred to as the hepatic falciform artery.^16–19^ The falciform ligament runs in continuity with the round ligament also known as the *ligamentum teres hepatis*, which results from the fibrous remnants of the embryonic umbilical vein and continues through the layers of the falciform towards the porta hepatis and anastomoses with branches of the liver before emptying into the inferior vena cava via the ductus venosus. The discussed cases in this report also confirm this vascular anatomy and perfusion of the posterior rectus sheath via the hepatic falciform artery through the use of ICG after harvesting the flap (see attached technique video).

### Posterior Rectus Sheath

The posterior rectus sheath has been used in liver transplantation for abdominal wall reconstruction after liver transplantation.^11,15,17^ In those case reports, the posterior rectus sheath flap is harvested in continuum with the falciform ligament attached to the donor liver. This fibrous sheath of dense connective tissue lined by parietal peritoneum is as a well-vascularized biologic flap from the donor that has the potential to provide sufficient and adequate strength to reconstruct the abdominal wall when a sizeable defect exists in the recipient. Similarly we use this robust biologic flap based off of the liver for sizable diaphragm defects, and the herein described procedure was performed under the supervision and guidance of the author of the first report of posterior rectus sheath use in liver transplantation (Dr L. Gottlieb).^15^

### Benefit of Rectus Sheath Repair

It is well established that among the many candidates for hiatal hernia repair in the USA, only 1–2% of all patients actually undergo surgical repair.^20–22^ While the reasons for this are complex (direct marketing to patients for PPIs, inadequate referral to surgeons, patient reluctance to undergo surgery in lieu of continued PPI use, etc.), surgeons are often faced with older patients with large hiatal hernia defects. The impetus to use prosthetic materials in such patients has been tempered by their associated complications and their lack of efficacy. The technique herein described has several advantages when surgeons are faced with such patients. Use of autologous vascularized tissue where the peritoneum is facing the operated stomach has a major advantage of reducing dense adhesion to the diaphragm should reoperative surgery be necessary. In addition, the dynamic tensile strength of autologous vascularized tissue may be more physiologic given the mobile and dynamic function of the diaphragm, the esophagus, and the stomach. Finally, avoiding placement of foreign material around the esophagus is a major advantage to avoid the risk of infection, stricture, or erosion with these prosthetics.

Our early outcomes show no signs of recurrence at 5-month follow-up after this repair of the hiatus with the posterior rectus sheath flap and no adverse effects of harvesting the posterior rectus sheath. Unfortunately, we cannot estimate these adverse effects from the previous use in transplantation as the rectus sheath was harvested from deceased donors. We hypothesized that by maintaining linea alba and linea semilunaris, there should not be a risk for abdominal wall hernias. However, the consequences of this defect in the unilateral posterior rectus sheath are unknown at this time. Longer-term follow-up with abdominal CT may better elucidate clinically relevant consequences and if there is a role for prophylactic mesh over the denuded rectus muscle.

With these concerns of abdominal wall disruption, successful application of this technique should include a thorough evaluation of the donor site preoperatively. Previous abdominal operations or disruption to the right upper abdominal wall as well as pre-existing abdominal wall defects may preclude the use of the right posterior rectus sheath. In these carefully selected patients, use of the robotic approach avoids disruption of the abdominal wall components and facilitates the technique of harvesting the posterior rectus sheath.

## Conclusions

Large paraesophageal hernia repairs and recurrent hiatal hernia repairs can be challenging given patient-related factors, the complex nature of the hernia defects, and the technical challenge of operating in a previously operated site. Although the long-term results of this procedure remains to be established, including its durability and safety, the report herein described suggests that this novel approach is a promising technique and is based on strong physiologic rationale.

## Supplementary Information

Below is the link to the electronic supplementary material.

Supplementary file1 (MP4 280774 KB)
